# Comparison of biochemical failure rates between permanent prostate brachytherapy and radical retropubic prostatectomy as a function of posttherapy PSA nadir plus ‘X’

**DOI:** 10.1186/1748-717X-9-171

**Published:** 2014-07-29

**Authors:** Kamran A Ahmed, Brian J Davis, Lance A Mynderse, Jeffrey M Slezak, Eric J Bergstralh, Torrence M Wilson, C Richard Choo

**Affiliations:** 1 Department of Radiation Oncology, Mayo Clinic, 200 First St SW, Rochester, MN 55905, USA; 2 Department of Physiology and Biomedical Engineering, Mayo Clinic, Rochester, MN, USA; 3 Department of Urology, Mayo Clinic, Rochester, MN, USA; 4 Division of Biomedical Statistics and Informatics, Mayo Clinic, Rochester, MN, USA; 5 Department of Research and Evaluation, Kaiser Permanente Southern California, Pasadena, California

**Keywords:** Biochemical failure, Brachytherapy, Prostate cancer, Prostatectomy, PSA

## Abstract

**Background:**

Prostate-specific antigen (PSA) nadir + 2 ng/mL, also known as the *Phoenix definition*, is the definition most commonly used to establish biochemical failure (BF) after external beam radiotherapy for prostate cancer management. The purpose of this study is to compare BF rates between permanent prostate brachytherapy (PPB) and radical retropubic prostatectomy (RRP) as a function of PSA nadir plus varying values of X and examine the associated implications.

**Methods and materials:**

We retrospectively searched for patients who underwent PPB or RRP at our institution between 1998 and 2004. Only primary patients not receiving androgen-deprivation therapy were included in the study. Three RRP patients were matched to each PPB patient on the basis of prognostic factors. BF rates were estimated for PSA nadirs + different values of X.

**Results:**

A total of 1,164 patients were used for analysis: 873 in the RRP group and 291 in the PPB group. Patients were equally matched by clinical stage, biopsy Gleason sum, primary Gleason grade, and pretherapy PSA value. Median follow-up was 3.1 years for RRP patients and 3.6 years in the PPB group (*P =* .01). Using PSA nadir + 0.1 ng/mL for the definition of BF, the 5-year BF rate was 16.3% for PPB patients and 13.5% for RRP patients (*P =* .007), whereas at nadir + 2 ng/mL or greater, the BF rates were less than 3% and were indistinguishable between PPB and RRP patients.

**Conclusions:**

In a cohort of well-matched patients who had prostatectomy or brachytherapy, we examined BF as a function of nadir + X, where X was treated as a continuous variable. As X increases from 0.1 to 2.0 ng/mL, the BF curves converge, and above 2.0 ng/mL they are essentially indistinguishable. The data presented are of interest as BF definitions continue to evolve.

## Background

Measuring serum prostate-specific antigen (PSA) is a recommended means for monitoring patients after radiation therapy [1]. A rising PSA level after treatment is frequently associated with subsequent clinical failure. Standardized definitions of biochemical failure (BF)—recurrence of PSA after treatment—have been developed to evaluate treatment efficacy and to detect clinical failure.

In 1997, the American Society for Therapeutic Radiology and Oncology (ASTRO) defined BF after external beam radiotherapy (EBRT) as 3 consecutive increases in PSA after treatment; the date of failure is set (backdated) at the midpoint between the time of PSA nadir and the first of the 3 increases [2]. This definition allowed for comparison of outcomes between treatment methods and institutions, but it had limitations. The process of backdating biases the Kaplan-Meier estimates of event-free survival and depends on the extent of follow-up [3,4]. The definition, known as the *ASTRO definition*, also was not optimized with respect to predicting clinical progression, survival, or therapeutic interventions. In 2006, using data from a multi-institutional database of patients treated with EBRT, ASTRO revised the definition of BF to be a PSA value 2 ng/mL greater than the patient’s absolute PSA nadir (“nadir + 2 ng/mL”) after EBRT [5]. This definition, known as the *Phoenix definition*, has been shown to have improved sensitivity and specificity in predicting subsequent clinical failure after radiotherapy as compared with the ASTRO definition and many other definitions [6-9]. Another advantage of the new definition is that it tends to limit the number of patients who are designated as having BF because it does not include those who merely experience a benign PSA “bounce” [10,11].

The recommended method of defining BF after radical retropubic prostatectomy (RRP) differs from that after radiation treatment. According to a recommendation by the American Urological Association Prostate Cancer Guideline Panel, the definition of BF after RRP should be an initial PSA level of 0.2 ng/mL or greater, with a second and identical confirmatory PSA level [12]. Thus, a PSA level above some minimal posttherapy level—nadir for radiotherapy, or undetectable for surgery—has become an important aspect of evaluating patients after both radiation and surgical treatment. However, the magnitude of the PSA value above nadir varies markedly between different therapies.

The purpose of this study was to compare rates of BF between well-matched cohorts of patients with prostate cancer who received two common primary treatment modalities, permanent prostate brachytherapy (PPB) or RRP, as a function of varying PSA nadir plus “X”. The results may aid in the comparison of different BF definitions across treatment modalities.

## Methods and materials

This study was approved by our institutional review board, and appropriate informed consent was obtained for review of patients’ medical records. By searching our prospectively maintained patient databases, we identified all patients who received PPB and RRP at our institution between January 1998 and December 2004. All patients were clinical stage T1c or greater and experienced a decrease in PSA or PSA nadir after primary therapy. The PPB group excluded salvage therapy patients. Prescription doses were consistent with those recommended by the American Brachytherapy Society Guidelines and include patients treated with I- 125 [13]. The RRP group excluded all patients who received neoadjuvant therapy or adjuvant radiation, as well as non–US residents unavailable for regular follow-up. Brachytherapy patients receiving neoadjuvant cytoreductive hormonal therapy were excluded, whereas high risk patients who received neoadjuvant and concomitant androgen deprivation but did not receive adjuvant treatment were included. Three RRP patients were matched to each PPB patient by a computerized matching process [14]. Patients were matched according to Gleason score on biopsy, disease stage, pretreatment PSA value, age, and year of procedure. Patients were followed up postoperatively with serum PSA measurement and digital rectal examination 3 to 4 months after treatment and then every 6 months for the first 5 years and annually thereafter.

Nadir was defined as the lowest PSA value achieved within 3 years of treatment. Subsequent BF was then defined based on PSA nadir plus “X,” where X varied from 0.1 to 5.0 ng/mL. BF was also considered to have occurred if there was no posttherapy nadir but the defined PSA increase occurred, if the patient received salvage treatment, or if androgen-deprivation therapy was implemented for an increasing PSA value after therapy.

We evaluated the 5-year Kaplan-Meier estimate of BF based on PSA nadir + X. Continuous variables were summarized as median and range or interquartile range; categorical variables were reported as number (percentage). Associations between continuous variables were assessed by Spearman’s correlation analysis. Conditional logistic regression was used to test differences between matched PPB and RRP patients. A 2-tailed *P <* .05 was considered statistically significant. All statistical analyses were performed using SAS 9.2 and JMP 7 (SAS Institute, Inc).

## Results

### Patient characteristics

**Figure 1:**
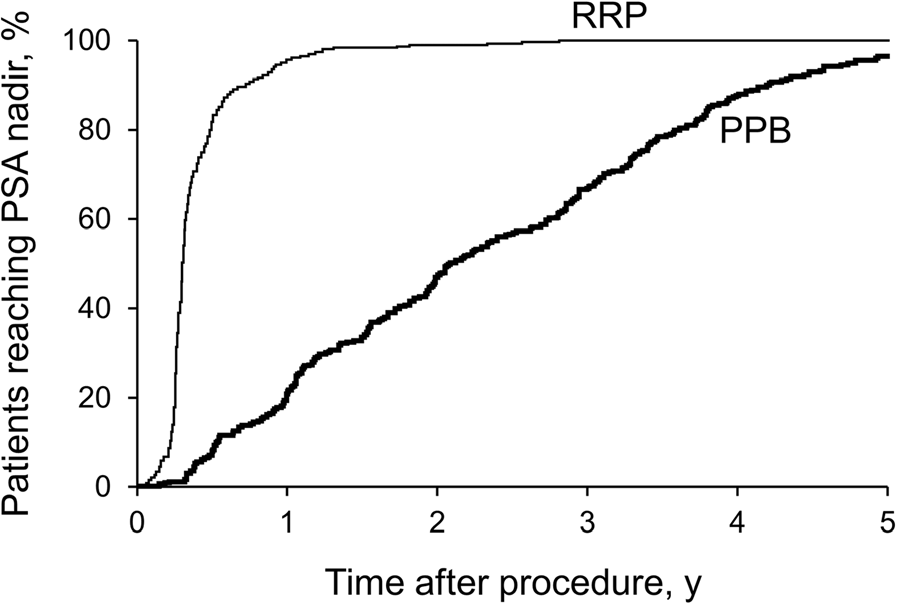
**Time to Prostate-Specific Antigen (PSA) Nadir.** Graph shows percentage of patients treated with permanent prostate brachytherapy (PPB; n = 291) and radical retropubic prostatectomy (RRP; n = 873) reaching PSA nadir by time after treatment.

A total of 518 patients received PPB and 5,821 patients received RRP at our institution during the study period. After applying inclusion and exclusion criteria, 1,164 matched patients were evaluated for this analysis: 873 RRP patients and 291 PPB patients. Patient characteristics are summarized in Table 1. Patients were equally matched by clinical stage (*P =* .47), biopsy Gleason score (*P =* .73), primary Gleason grade (*P =* .25, data not shown), and pretherapy PSA value (*P =* .95). Median age was similar in the PPB and RRP groups (69 vs 68 years; *P =* .11). Median follow-up was 3.6 years for RRP patients and 3.1 years in the PPB group (*P =* .008). Median time to PSA nadir was 3 months after RRP and 2.4 years after PPB (Figure 1).

**Table 1:** Patient characteristics

	**Patient Group**		
**Characteristic**	**PPB (n = 291)**	**RRP (n = 873)**	***P*** **Value**
Age, y			.11
Median range	69 (41–80)	68 (43–80)	
Mean (SD)	67.2 (6.7)	66.6 (6.21)	
Clinical stage			.47
T1	204 (70.1%)	592 (67.8%)	
T2	87 (29.9%)	281 (32.2%)	
Biopsy Gleason score			.73
6	235 (80.8%)	722 (82.7%)	
7	36 (12.4%)	104 (11.9%)	
≥8	20 (6.8%)	47 (5.4%)	
Pretherapy PSA value, ng/mL			.95
Median (IQR)	5.7 (4.1-7.7)	5.8 (4.1-7.6)	
Range	0.6-17.5	0.6-23.1	
Time from therapy to death or last follow-up, y			.008
Median	3.1	3.6	
Maximum	6.9	7.5	
Systemic progression	4 (1.4%)	4 (0.5%)	.10
Biopsy-proven local recurrence	1 (0.3%)	5 (0.6%)	.63

Abbreviations: IQR, interquartile range; PPB, permanent prostate brachytherapy; PSA, prostate-specific antigen; RRP, radical retropubic prostatectomy.

Brachytherapy implant characteristics for the PPB patients are summarized in Table 2. A total of 273 patients underwent prostate brachytherapy monotherapy and 28 underwent combined treatment with external beam radiotherapy. All twenty patients with Gleason 8 received combined treatment and 8 of 36 patients with Gleason 7 cancer received combined treatment. Of those patients undergoing monotherapy, median prostate volume was 39.7 mL, and median implanted mCi was 42.6. Four PPB patients (1.4%) and 4 RRP patients (0.5%) had systemic progression (*P =* .10) (Table 1). There were no differences in local recurrence between the RRP and PPB cohorts: 0.6% vs 0.3% (*P =* .63).

**Table 2:** Dosimetry characteristics for patients undergoing monotherapy (I-125) in PPB Group (n = 273)

**Characteristic**	**Median (IQR)**
Prostate volume, mL	39.7 (31–47)
mCi implanted	42.6 (36.4-48.2)
Prostate D90, Gy	158 (146–174)
Prostate V100,%	94.7 (90.3-97.8)
Rectal V100, mL	0.28 (0.01-1.08)

Abbreviations: D90, isodose enclosing 90% of the prostate; IQR, interquartile range; PPB, permanent prostate brachytherapy; V100, target volume receiving 100% of the prescribed dose.

**Figure 2:**
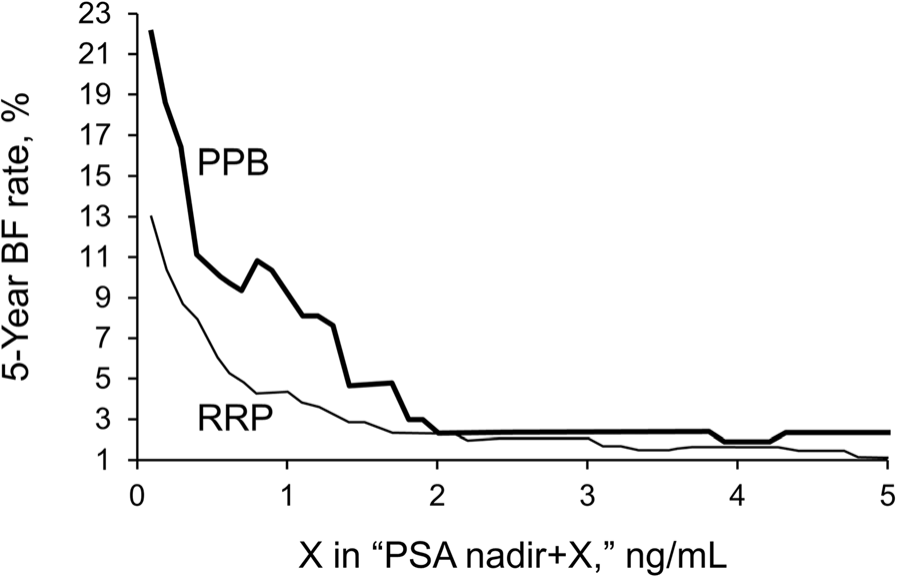
**Biochemical Failure (BF) as a Function of Prostate-Specific Antigen (PSA) Nadir + X.** Graph shows Kaplan-Meier estimated 5-year rate of BF in patients treated with permanent prostate brachytherapy (PPB; n = 291) and radical retropubic prostatectomy (RRP; n = 873) for different definitions of PSA nadir + X. The X-axis shows the value of “X” ng/mL PSA in the equation “PSA nadir + X.”

We compared the rates of BF by PSA nadir plus varying values of X between the RRP and PPB groups (Figure 2). At nadir + 0.1 ng/mL, the 5-year BF estimate (SE) for PPB patients was 16.3% (2.3%) and 13.5% (1.5%) for RRP patients (*P =* .007). At nadir + 1.0 ng/mL, 5-year BF was 9.1% (2.1%) versus 4.9% (1.1%) for the PPB and RRP groups, respectively (*P =* .09). BF rates were less than 3% at nadir + 2 ng/mL or greater and were indistinguishable between PPB and RRP patients (*P =* NS). Using two different definitions of BF based on treatment modality, nadir + 2 ng/mL or greater for PPB patients and nadir + 0.1 ng/mL or greater for RRP patients, 5-year BF rates were less than 3% and 13.5%, respectively.

## Discussion

In this study, we applied the most commonly used and accepted definition of BF in prostate cancer treated with EBRT to a large group of patients treated with PPB or RRP. The patients in the two groups were matched by prognostic factors of Gleason score by biopsy, stage, and PSA value, and year of procedure. We analyzed and compared rates of BF between patients treated with PPB and RRP as a function of a varying PSA nadir plus X. The aims of the study were 1) to examine differences in BF rates in groups of patients with similar prognostic factors and similar clinical outcome with respect to systemic progression, and 2) to provide data to determine if a similar PSA definition for EBRT, brachytherapy, and surgery is tenable. To our knowledge, this is the first study to provide such comparative data and analysis.

Most of the patients in this study had low-risk disease. Studies have demonstrated similar outcomes in biochemical progression–free survival between men treated with RRP and PPB for low-risk disease [15-17]. A recent study by Arvold et al. [18] of 5,760 men with low-risk prostate cancer and 3,079 men with intermediate-risk prostate cancer found no significant difference in the risk of prostate cancer–specific mortality between these groups after RRP or PPB. In our study, there were no significant differences in systemic progression or biopsy-proven local recurrence between patients treated with PPB or RRP.

Although definitions of BF after EBRT have been well studied, these definitions were not intended to be applied to patients treated with PPB or RRP; there is no accepted BF definition that can be applied universally to patients treated with PPB, RRP, and EBRT. For this reason, it is also of interest to examine how definitions of BF compare to one another to better assess outcomes between treatment modalities.

The most commonly used definition of BF after EBRT, the Phoenix definition, evolved from the previous ASTRO definition [2]. Several authors have noted limitations with the ASTRO definition, including not being linked to survival, clinical progression, or interventions [4,19,20]. The definition was also very sensitive to follow-up and led to false-positives from benign PSA bounces [10,11]. Several other PSA definitions were suggested, one of which was PSA doubling time as a predictor of failure after EBRT [11,21-23]. In a study of 4,839 patients treated between 1986 and 1995 at 9 academic institutions, Thames et al. [4] found additional definitions to be superior to the ASTRO definition in terms of sensitivity, specificity, positive and negative predictive values, and risk of clinical failure after BF. These definitions included two increases of at least 0.5 ng/mL, PSA level at or greater than the current nadir plus 2 or 3 ng/mL, and PSA level at or greater than the absolute nadir plus 2 ng/mL. The study found the Phoenix definition to have better sensitivity (.67) and specificity (.84) than the ASTRO definition (.61 and .80) for predicting clinical failure.

Since nadir + 2 ng/mL is the most common definition of BF after EBRT, it is often used in series of patients treated with PPB [24,25]. Kuban et al. [26] analyzed 2,693 patients treated with PPB and found the nadir + 2 ng/mL definition to provide the best surrogate for failure throughout the entire follow-up period, similar to patients treated with EBRT. The nadir + 2 ng/mL definition was more sensitive and specific than PSA doubling time or a certain number of PSA increases. Although the intent of our study was not to propose nadir + 2 ng/mL as a definition of BF for both PPB and RRP patients, 5-year BF rates in this study become similar for the two cohorts using a nadir + 2 ng/mL or greater definition of BF.

After RRP, PSA levels should theoretically become undetectable because the source of PSA, the prostate, is completely excised; therefore, a measureable PSA on follow-up has traditionally been assumed to indicate BF after surgery. Stephenson et al. [27] evaluated 10 definitions of BF in 3,125 patients who underwent prostatectomy. The study found that a PSA of at least 0.4 ng/mL followed by another increase best explained metastatic progression. In contrast, the Phoenix definition has not been validated in surgically treated patients and has been found to delay the diagnosis of BF. In a study of 2,570 patients who underwent RRP, Nielsen et al. [28] found that the 5-year biochemical control rates for a definition of nadir + 0.2 ng/mL or more were similar to the 10-year biochemical control rates using the nadir + 2 ng/mL definition. After reviewing the literature from 1991 to early 2004, the American Urological Association recommended using the nadir + 0.2 ng/mL definition to define BF after surgery [12].

Analyzing BF as nadir + X allows X to be a continuous variable. In the present analysis, as PSA continues to increase above nadir, there is less of a difference in BF rates between RRP and PPB patients. At the ASTRO definition of nadir + 2 ng/mL, the curves assessing BF between our matched PPB and RRP cohorts become indistinguishable. As PSA continues to increase above nadir, the likelihood of biochemical and clinical failure increases, indicating disease progression.

As demonstrated in our study and in the detailed study by Thames et al. [4], there is a fine line in balancing sensitivity and specificity for choosing a definition for BF. If BF is defined as a minimal increase in PSA after nadir, sensitivity of BF increases. At a low value of X, a larger patient population meets the classification for BF, as noted in Figure 2. By using a minimal increase in PSA after nadir to define BF, emphasis is placed on treatment and better recognition of local recurrence. However, specificity increases when using a larger increase in PSA after nadir to define BF, and the likelihood that these patients had local, regional, or distant failure is increased. Choosing a definition that balances specificity and sensitivity was a question faced by the ASTRO consensus panel when the Phoenix definition was established. In our study, we find similarly indistinguishable outcomes as X continues to increase above nadir + 2 ng/mL.Although the study contains a large number of patients it might be expected that a smooth downward trend with increasing values of X would be observed in Figure 2. This is most evident in the PPB curve and the increase between nadir + 0.8 ng/mL and nadir + 0.9 ng/mL. The number of patients at a given nadir + X level may vary such that when a failure event occurs, the overall failure rate at a higher nadir + X may appear greater than for smaller values of X due to differences in duration of follow up and patients and number censored at a given nadir + X level. Nevertheless, the general trend downward as X increases remains evident and is consistent with prior studies analyzing PSA failure and EBRT [Thames].

Our study had several limitations. First, our analysis is subject to many of the limitations common to retrospective analyses. It is also a single-institution study, which may introduce selection bias. Although our cohorts of PPB and RRP patients were well matched, some statistical differences were noted in length of maximum follow-up. Because surgical treatment is relatively more established in our institution compared with brachytherapy, the RRP group had a longer follow-up period at the time of analysis. However, this is unlikely to have affected our analysis of BF between the two techniques. In addition, although we observed no significant differences in systemic progression between the two cohorts, the small number of total events (n = 8) limits the power for such comparisons and the ability to link nadir + X with later clinical events.

## Conclusion

In a cohort of well-matched patients who underwent either RRP or PPB and had similar local recurrence and systemic failure rates, we examined BF as a function of PSA nadir + X, where X was treated as a continuous variable. As X increases from 0.1 to 2.0 ng/mL, the 5-year Kaplan-Meier BF estimate curves for the two groups converge, and above 2.0 ng/mL they are essentially indistinguishable. These data and analysis are of interest in examining means by which to compare biochemical failure between differing treatment modalities.

## Competing interests

The authors declare that they have no competing interests.

## Authors’ contributions

KAA carried out the design of the study, data retrieval, statistical analysis, and drafted the manuscript. CAM, EJB, TMN, CRC carried out a critical review of the manuscript. JMS carried out statistical analysis of the manuscript. BJD conceived of the study, and participated in its design and coordination. All authors read and approved the final manuscript.
